# Complex Lower Back Pain: A Case Report

**DOI:** 10.7759/cureus.99137

**Published:** 2025-12-13

**Authors:** Jéssica Ribeiro, Luís Cardoso Rocha, Ana Soares, José Augusto Santos

**Affiliations:** 1 Family Medicine, Family Health Unit Ponte Velha, Local Health Unit Médio Ave, Santo Tirso, PRT

**Keywords:** acute illness consultation, acute myelopathy, clinical case report, diabetic neuropathies, family medicine, lower back pain (lbp), polineuropathy

## Abstract

Lower back pain is a common primary care symptom with musculoskeletal, neurological, neoplastic, infectious, or inflammatory causes. It remains the leading cause of years lived with disability, and it is linked to modifiable factors such as occupational exposure, smoking, and high body mass index (BMI), highlighting the need for preventive, lifestyle-focused strategies. To illustrate these challenges, a 60-year-old man is presented with a 20-year history of type 2 diabetes and a background of alcohol use disorder and suboptimal therapeutic compliance, resulting in poor glycemic control. He presented with low back pain radiating to both lower limbs, with a five-day evolution, exhibiting a mechanical pattern, without identifiable relieving or aggravating factors, which progressively progressed to tetraparesis. Following a comprehensive, multisystem evaluation conducted by both the primary care physician and hospital specialists, the etiology was determined to be multifactorial. Cervical spondylotic compressive myelopathy was confirmed through cervical MRI, leading to the indication for surgical intervention. The mixed sensorimotor polyneuropathy with axonal predominance was diagnosed via electromyography and nerve conduction studies, consistent with combined diabetic and toxic etiologies. The patient achieved partial symptom remission; however, significant limitations in activities of daily living persisted, ultimately requiring disability retirement, with substantial psychological and social impact. Grounded in the principles of family medicine, this case illustrates the crucial role of the general practitioner: guiding the evaluation of a common symptom through individualised care; obtaining a medical history that raised diagnostic suspicion; and managing a complex patient with a holistic approach that included physiotherapy, optimisation of diabetes control, medication review, and mental health support. The general practitioner also addressed cardiovascular risk factors to delay or prevent disease progression and promoted sustained healthy lifestyle changes.

## Introduction

Lower back pain (LBP) continues to represent one of the greatest global public health challenges, remaining the leading cause of years lived with disability across all age groups. Recent data from the Global Burden of Disease 2021 estimate that, in 2020, more than 619 million people were affected by low back pain, with projections indicating an increase to 843 million by 2050, driven mainly by population ageing and overall population growth [1]. The lifetime incidence of LBP is remarkably high, affecting up to 84% of the general population and carrying a high recurrence rate [2,3]. Although age-standardized prevalence and disability rates have shown a slight decline over the past three decades, the absolute burden of the condition continues to rise. Higher prevalence is observed in women and in older adults, with a peak around 85 years of age [1]. Approximately 38.8% of disability associated with low back pain is attributable to modifiable factors-namely, occupational exposures, smoking, and high body mass index-highlighting the importance of preventive strategies and early interventions focused on lifestyle modification and improvements in workplace conditions [1].

LBP can be acute (less than two to four weeks), subacute (four to 12 weeks), or chronic (more than 12 weeks) [2]. Acute LBP accounts for a substantial proportion of medical visits and is the second most common reason for appointments in general practice. Chronic LBP is the eighth most frequent reason for consultation [2]. This condition also has a considerable impact on occupational health, due to its high prevalence and substantial burden on working-age individuals, with productivity losses and lost workdays. Additionally, LBP might force workers to retire prematurely [1].

Clinical features that require urgent evaluation in patients with low back pain, such as progressive neurological deficits, saddle anesthesia, bladder or bowel dysfunction, unexplained weight loss, or history of trauma or malignancy, should be evaluated to identify patients at risk of serious spinal or neurological pathology and to guide timely referral and further diagnostic work-up [4]. Recognition of these warning signs is essential in general practice, as sensory-motor neurological symptoms such as decreased muscle strength and paresthesia may indicate involvement of the central nervous system (CNS) or the peripheral nervous system (PNS). Sensory-motor neurological symptoms are also frequent in general practice consultations [5]. Lesions of the sensory pathways can produce changes in tactile, nociceptive, thermal, proprioceptive, and vibratory sensitivity. Lesions of the motor pathway lead to decreased muscle strength and changes in muscle tone, and in some cases, involuntary muscle activity [3].

The clinical presentation of a lesion in the CNS, particularly the spinal cord, depends on the affected section and may result in spinal cord syndromes [6]. At the PNS level, lesions are classified according to the site of involvement: neuropathies (affecting peripheral nerves), radiculopathies (affecting one or more nerve roots), and plexopathies (affecting the plexus and its associated nerves) [7]. 

The joint recommendations of the European Academy of Neurology and the Peripheral Nerve Society (EFNS/PNS), often referred to as the EFNS/PNS criteria, propose a classification based on levels of diagnostic certainty (possible, probable, definite), integrating symptoms, clinical signs, electrophysiological studies, and, when relevant, small-fiber assessment tests. Thus, the EFNS/PNS criteria constitute an internationally accepted reference for defining and stratifying peripheral neuropathy, allowing differentiation between axonal and demyelinating patterns, assessment of dysfunction severity, and standardization of classification across clinical and research studies [8].

In clinical practice, the temporal profile of symptom evolution constitutes an important diagnostic clue, helping to narrow the differential diagnosis and guide further evaluation. Therefore, neuropathies can be classified according to symptom duration as acute, subacute, or chronic [5,9]. In acute neuropathy, there are autoimmune causes (Guillain-Barré syndrome), metabolic causes (uremic neuropathy), toxic causes (alcohol), critically ill patients, and diabetic amyotrophy (Bruns-Garland syndrome) [5,9]. Toxic (alcohol, drugs) and metabolic etiology (vitamin deficit) may induce subacute neuropathy [5,9]. Chronic neuropathy, usually defined as symptoms that last more than eight weeks, can be caused by hereditary diseases, metabolic diseases (diabetes mellitus, chronic renal failure, hypothyroidism), HIV, toxic causes (alcohol), or chronic inflammatory demyelinating polyradiculoneuropathy [5].

Diabetic neuropathy is one of the most prevalent and disabling chronic complications of diabetes, which can affect 25% to 50% of patients with diabetes, resulting from progressive damage to the peripheral nervous system associated with prolonged metabolic exposure [5,10]. The American Diabetes Association (ADA) Standards of Care describe diabetic neuropathies as a heterogeneous group of peripheral nervous system disorders, characterized by symptoms and/or signs of nerve dysfunction that arise in the context of diabetes, after exclusion of other potential causes [11]. In the presence of abnormal metabolic factors, the structure and function of various components of the nervous system can be compromised. Furthermore, irregularities in the insulin signaling pathway can inhibit axon repair and promote cell apoptosis [10]. Diabetic neuropathy is classified into different syndromes, based on the symptoms and signs that characterize them and the component of the peripheral nervous system affected. The most common type of diabetic neuropathy is a distal symmetric polyneuropathy, which is characterized by progressive loss of distal sensitivity correlated with loss of sensory axons, followed, in severe cases, by motor weakness and loss of motor axons. A typical stocking-glove distribution occurs [10].

Nevertheless, diabetic neuropathy can affect thoracic and lumbar nerve roots (polyradiculopathies) or have autonomic involvement [10]. Rarely, thoracic polyradiculopathy can occur, with severe abdominal pain and, in some cases, chest and upper limb involvement. However, the most common type of polyradiculopathy is the diabetic amyotrophy, also known as Bruns-Garland syndrome, diabetic myelopathy, or diabetic neuropathy of the lumbosacral radiculoplexus. This refers to a group of peripheral nerve disorders characterized by anatomical location involving the lumbosacral plexus. It causes neuropathic pain, weakness, progressive proximal atrophy, and weight loss. It occurs in approximately 0.8% of all patients with diabetes and most frequently affects men, generally over 50 years of age. It may occur as a complication of a pre-diabetic state or as a result of an acute reduction in blood glucose/tight control in a patient with chronic hyperglycemia [9,12,13].

## Case presentation

The patient is a 60-year-old widowed man, currently residing with his partner in northern Portugal. He is employed as a furnace operator and has attained less than a fourth-grade level of education. He is independent in activities of daily living (ADL) and demonstrates preserved cognitive function.

His personal medical history includes 20 years of poorly controlled type 2 diabetes mellitus, diabetic retinopathy, grade I obesity, arterial hypertension, dyslipidemia, hyperuricemia, benign prostatic hyperplasia, and erectile dysfunction. He currently drinks one glass of wine per day, although he has a history of alcohol use disorder, and he is a former smoker. He reports no other toxic exposures and no relevant family history. He is receiving pharmacological treatment for the conditions mentioned above. For his diabetes, he takes metformin, empagliflozin, human insulin, and dulaglutide. Dulaglutide was initiated in February 2022, since which time he has achieved satisfactory metabolic control.

In late February 2023, the patient presented to the primary health care unit for an acute illness appointment, with lower back pain radiating bilaterally to the lower extremities, without other associated symptoms such as fever, progressive neurological deficits, saddle anesthesia, bladder or bowel dysfunction, unexplained weight loss, or history of trauma or malignancy. On physical examination, the patient exhibited a painful gait, claudication, a positive bilateral straight leg raise test (Lasègue's sign), and severe left paravertebral muscle spasm. He was prescribed cyclobenzaprine 10 mg twice daily and diclofenac 75 mg twice daily for five days. A temporary disability certificate was issued to justify his work absence.

The following week, the patient returned for a follow-up acute illness appointment due to persistent lower back pain radiating to his lower limbs. He additionally reported the onset of lower limb weakness and right arm pain. The previously prescribed treatment did not lead to clinical improvement. The therapeutic plan was adjusted to continue cyclobenzaprine, request a lumbar CT scan, and extend the temporary disability certificate.

Two weeks later, the patient revisited the primary health care unit with persistent complaints, at this point accompanied by the onset of paresthesia in the upper limbs. The lumbar CT scan revealed a sinistroconvex scoliosis, L5 anterolisthesis secondary to L5 isthmic lysis, and marked degenerative changes in the interapophyseal and, more significantly, the spondylodiscal joints, with potential pluriradicular compression. He was medicated with tramadol 37.5 mg + paracetamol 325 mg and pregabalin 75 mg, and subsequently referred for a consultation in the Orthopedics department at the local reference hospital.

In April 2023, the patient attended a scheduled consultation with his family physician, presenting with a seven-week history of insidious-onset, progressive tetraparesis. His symptoms included gait disturbance with neurogenic claudication, requiring the use of crutches for ambulation. Functional assessment revealed marked gait impairment, with a Functional Ambulation Classification (FAC) score of 3, indicating the need for continuous physical assistance. He also displayed distal upper-limb pain with paresthesia, cold sensation, and progressive loss of strength. At this stage, the patient was unable to perform basic ADLs independently, reflected in a Barthel Index score of 65/100. He was initiated on Cyanocobalamin + Pyridoxine + Thiamine (0.2 mg + 200 mg + 100 mg) and maintained on his previous medication regimen. A referral to the Neurology department was requested with a "very urgent" priority. Targeted laboratory studies were performed, with findings including an elevated erythrocyte sedimentation rate (ESR) of 37 mm/h. Other requested parameters [complete blood count, folic acid, vitamin B12, C-reactive protein, thyroid-stimulating hormone (TSH), and electrolytes] were within normal limits.

Two weeks later, the patient revisited his primary care physician, reporting an exacerbation of pain, subjective strength loss, and paresthesias in the upper limbs, which now impaired his ability to use crutches. On physical examination, strength loss was classified by the manual muscle testing (MMT) grading scale: right shoulder abduction and right wrist flexion were graded at G4/5, right hip flexion was graded at G4/5, and left hip flexion at G5-/5. It was noted that the neurology consultation request had been denied with the recommendation to obtain an urgent cervical CT scan, stating that "if there is no evidence of cervical myelopathy and the progression continues, evaluation in the emergency department is warranted".

**Figure 1:**
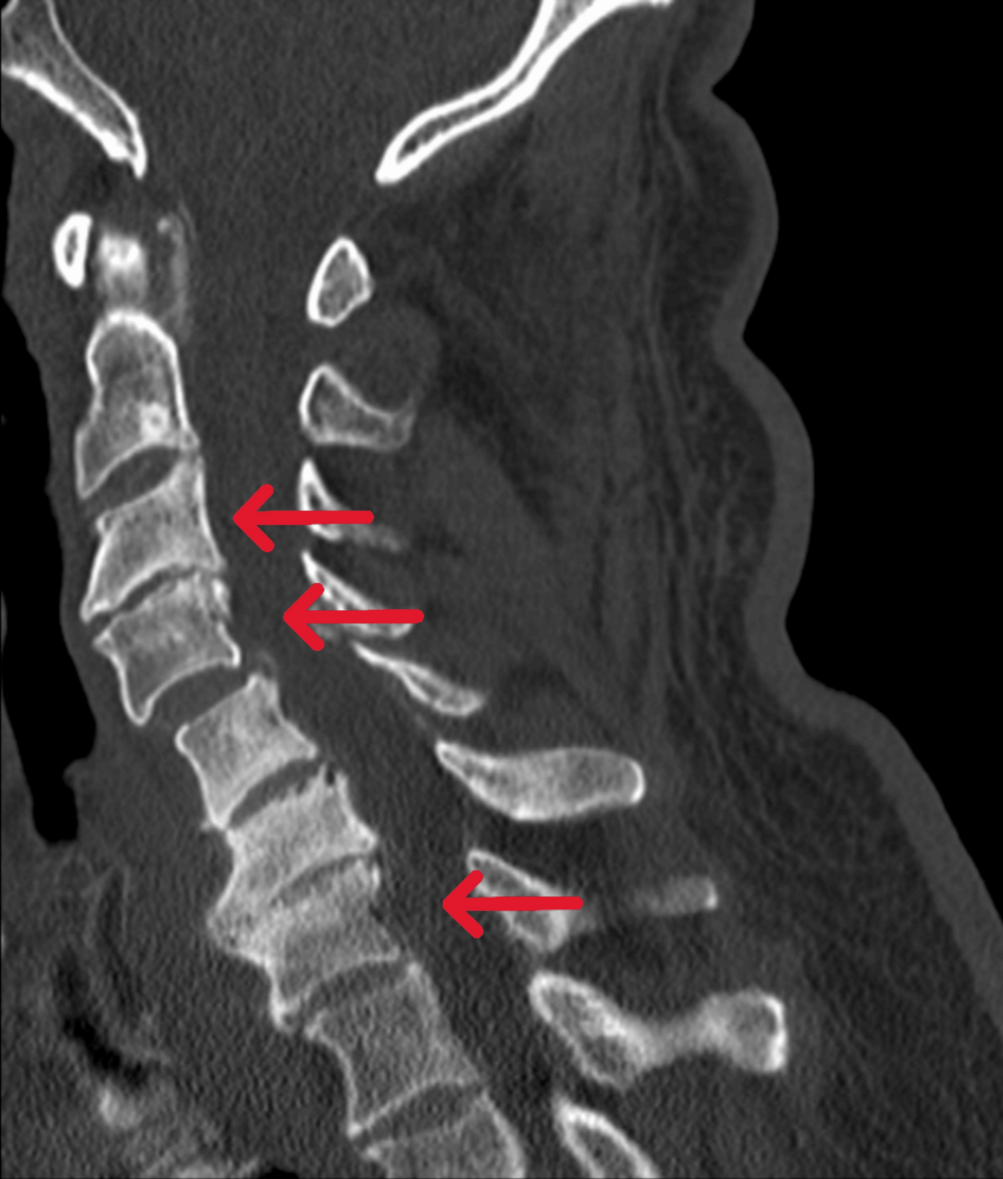
Cervical CT scan Cervical CT scan (sagittal plane) depicting C3 retrolisthesis, C4, and C7 anterolisthesis.

An urgent cervical CT and electromyography (EMG) of both the upper and lower limbs were ordered and completed in the subsequent days. The cervical CT scan (Figure 1) revealed dextroconvex scoliosis, C3 retrolisthesis, C4 and C7 anterolisthesis, and cervical discovertebral degenerative changes with potential compromise of the left C4 and C8 nerve roots at C3-C4 and C7-D1, as well as the right C5 root at C4-C5. EMG showed slowed motor conduction velocities, prolonged F-wave latencies, and patchy conduction block, suggesting a possible demyelinating component within EFNS/PNS criteria. However, axonal features were limited at that stage. These findings were consistent with a severe acute/subacute demyelinating polyneuropathy, more pronounced in the lower extremities, suggesting the need to rule out sequelae of Guillain-Barré syndrome. Due to the progression of symptoms, the patient's increasing dependency, and diagnostic findings indicative of a demyelinating disease, he was referred to the Emergency Department for a neurological evaluation.

A neurological examination was performed in the emergency department, revealing the following findings: Right shoulder abduction and right wrist flexion were graded at G4/5 by the MMT Grading Scale. The remaining segments were G5/5. Right hip flexion was graded at G4-/5 and left hip flexion at G5-/5. The remaining segments were G5/5. Bilateral finger flexor reflexes were brisk. The right patellar reflex was brisker than the left with a widened stimulation area. Achilles reflexes were absent bilaterally.

Other findings included a positive left Hoffmann's sign. Pain sensation was intact in the trunk and limbs, with no discernible sensory level. There were some proprioceptive errors in all four limbs, compounded by paresthesias that made interpretation difficult. Reduced vibratory sensation (hypo-pallesthesia) was present up to the knee level bilaterally. The finger-to-nose test was performed without ataxia. Gait required unilateral support and demonstrated right lower limb claudication.

A diagnosis of progressive paraparesis was presumed, with a several-month evolution that significantly impacted the patient's functionality. A significant deterioration was observed: whereas he had previously exhibited a normal gait, at present, he required bilateral crutch support. The presence of upper motor neuron signs led to the hypothesis of myelopathy.

The findings in the cervical CT scan were discussed with the neurosurgery team. It was determined there were no unequivocal signs of spinal cord compression, despite the EMG report suggesting a demyelinating polyneuropathy/Guillain-Barré syndrome. The clinical picture had progressed for more than three months, and there were no clear signs of lower motor neuron disease except for the abolished Achilles reflexes. 

**Figure 2:**
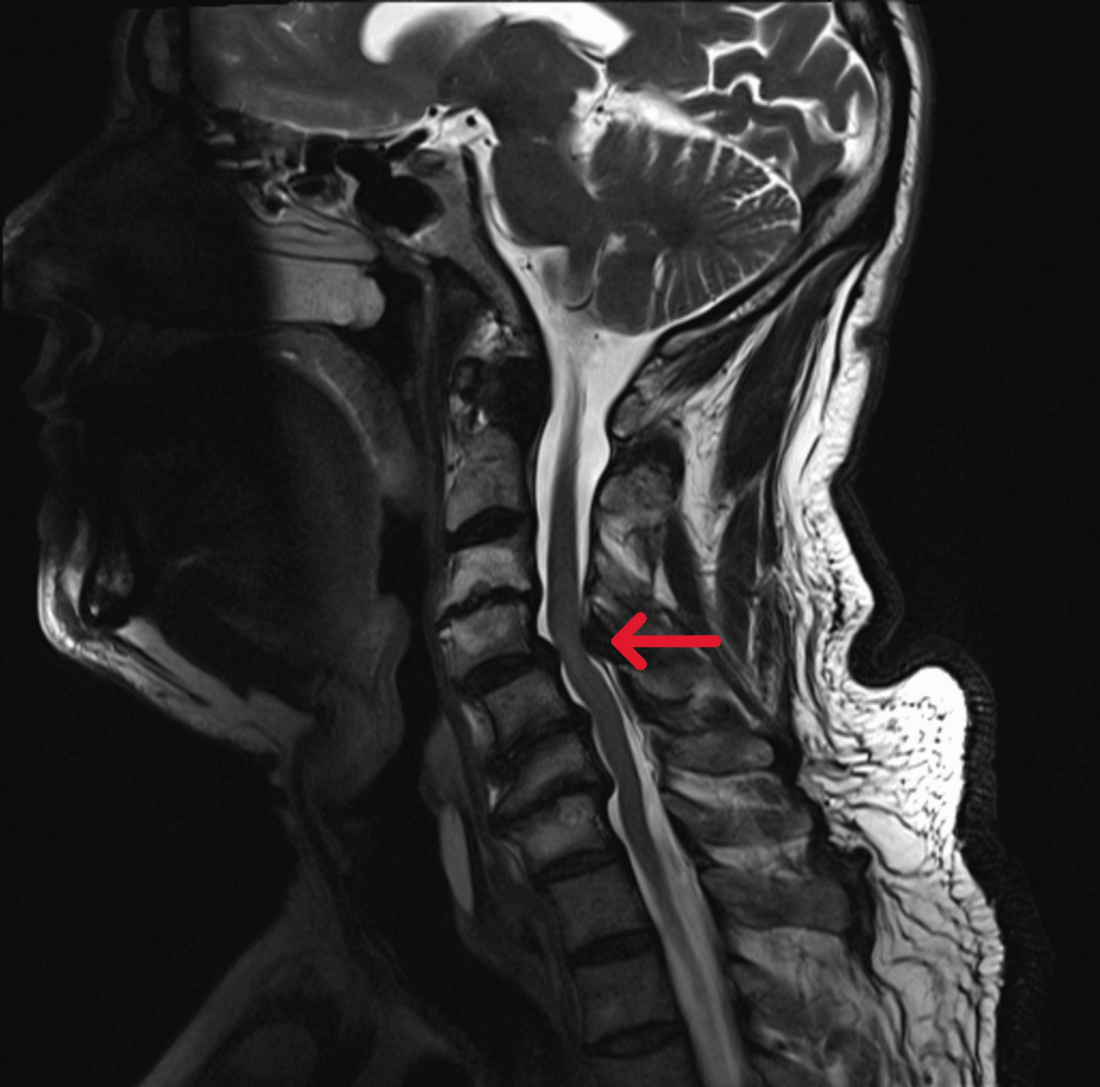
Cervical MRI Cervical MRI (T2-TSE, sagittal plane) showing reduced anteroposterior diameter of the spinal canal with indentation of the spinal cord at the C4-C5 level.

**Figure 3:**
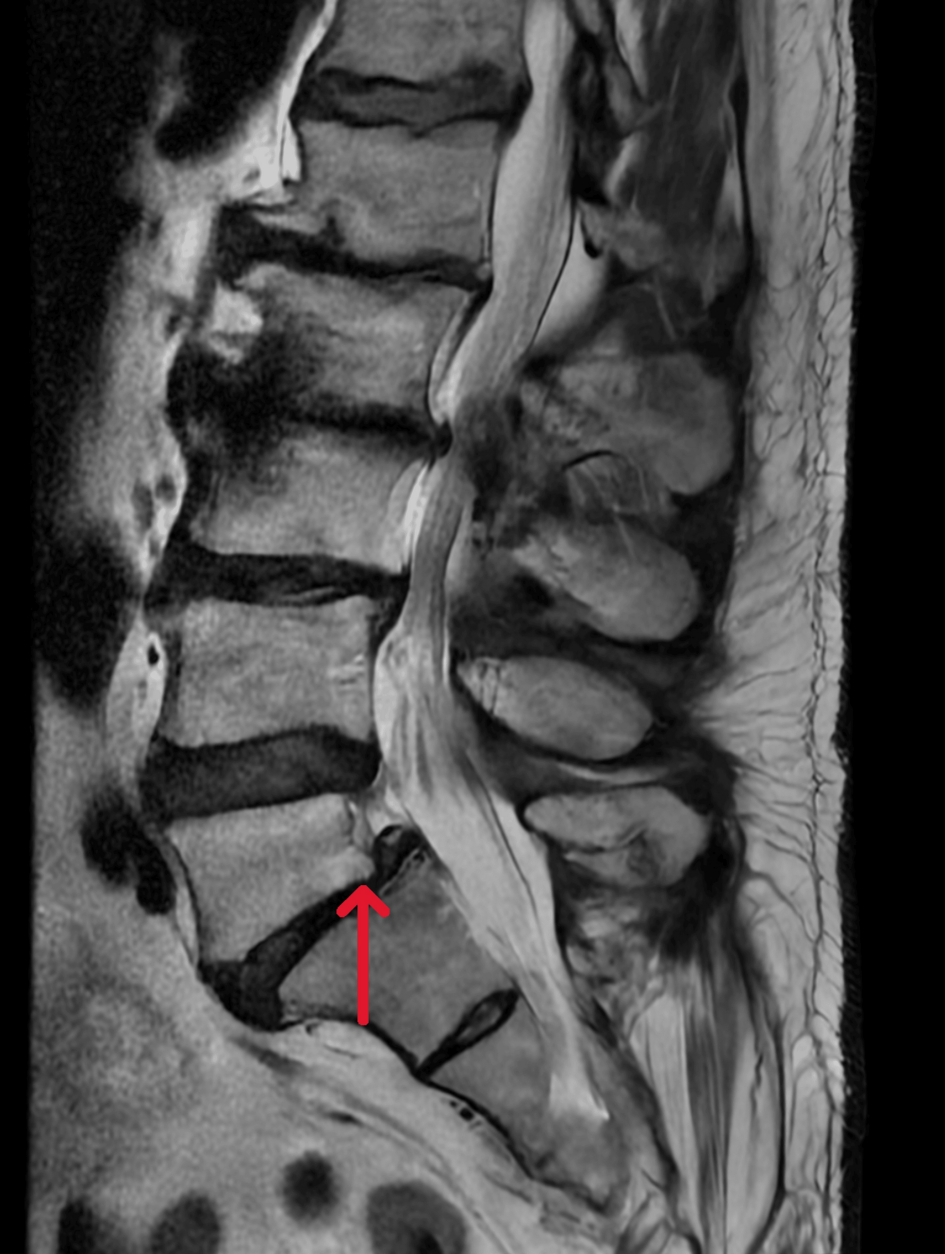
Lumbar MRI Lumbar MRI (T2-FSE, sagittal plane) showing L5 anterolisthesis.

The patient was admitted to the neurology department for further investigation. During the inpatient stay, a cervical magnetic resonance imaging (MRI) was performed (Figure 2), which stated a broad-based posterior disc pseudobulge at the C4-C5 level and thickening of the ligamenta flava that reduced the anteroposterior diameter of the spinal canal with indentation of the spinal cord, defining T2TSE/STIR cord hyperintensity, suggestive of probable spinal cord signal change/myelopathy. These findings occur within multilevel degenerative changes, disc protrusion, and uncovertebral arthrosis that are causing probable nerve root compression, primarily on the left side from C3 through T1. A lumbar MRI was also obtained (Figure 3), which revealed Grade I anterolisthesis in L5 and marked disc pseudo-protusion in L5-S1, resulting in significant narrowing that is likely compressing both L5 nerve roots. Additionally, active inflammatory changes (Modic type I) at L2-L3, widespread interapophyseal arthrosis, and bilateral posterior ligamentous thickening are causing impingement of the L2, L3, and L4 nerves at multiple levels. Given the diagnostic overlap between Guillain-Barré syndrome (GBS), chronic inflammatory demyelinating polyneuropathy (CIDP), early diabetic neuropathy, and mixed toxic-metabolic neuropathies, a repeat EMG was performed. This second study demonstrated a moderate to severe chronic sensorimotor polyneuropathy with clear axonal predominance, particularly in the lower limbs, aligning more closely with diabetic neuropathy and possible toxic-alcoholic contribution.

**Figure 4:**
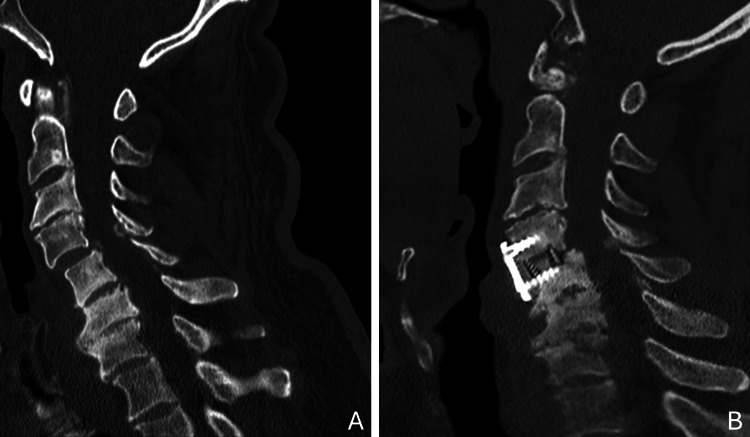
Cervical CT scan Cervical CT scan (sagittal plane) pre-arthrodesis (A) and post-arthrodesis (B)

The final diagnoses were established as tetraparesis secondary to compressive cervical myelopathy and a sensorimotor polyneuropathy of predominantly axonal etiology, likely a mixed picture of diabetic neuropathy with a possible toxic-alcoholic contribution. The patient was proposed for surgical correction via a cervical plate for arthrodesis, which was performed during a subsequent elective admission and proceeded without complications. The surgical procedure focused on the treatment of cervical myelopathy and C4-C5 instability via C4-C5 microdiscectomy, removal of the broad-based disco-osteophytic protrusion, medullary decompression, C4-C5 interbody arthrodesis using a Polyetheretherketone (PEEK) Cornerstone cage (Medtronic Sofamor Danek, Inc., Memphis, Tennessee, USA) 14x11x5 filled with nanostim hydroxyapatite matrix, and anterior fixation with a Medtronic Zevo plate - 21 mm (Medtronic Sofamor Danek, Inc., Memphis, Tennessee, USA). Fluoroscopy was used. Figure 4 presents cervical CT scans acquired pre-arthrodesis (Figure 4A) and post-arthrodesis (Figure 4B).

The patient's follow-up was continued by his family doctor. In addition to managing the remaining comorbidities and therapeutic regimen, as well as controlling cardiovascular risk factors (CVRFs), the general physician recommended physiotherapy treatments for rehabilitation and maintenance of the temporary disability certificate.

In subsequent neurosurgery consultations, it was reported that the patient regained his gait and maintained a clear benefit from the surgery, specifically in his dexterity and a considerable gain in autonomy. Nevertheless, the family physician reports that the patient continues to experience lower back pain, although less intensely, along with residual neuropathic pain and paresthesia in his upper and lower limbs. The patient continued to use crutches for ambulation. These conditions became of chronic significance and led the patient to require evaluation to determine permanent disability to perform his job and subsequent disability retirement, which was granted. 

The family physician also reported worsening in psychological and mental health during the year that followed the beginning of the clinical scenario, due to the onset and progressive urge of symptoms, transient loss of capacity to perform ADLs, chronicity of symptoms, and disability retirement. The patient presented with signs of anxiety and depression. There was no need for a psychologist or psychiatric evaluation or medication for such symptoms, as these were properly assessed by the family doctor and improved. The patient also highlighted the importance of his partner's support.

Table 1 depicts the key clinical milestones during the entire clinical course.

**Table 1:** Key clinical milestones during the clinical course

Date	Milestone
2023-02-24	Acute illness consultation: lower back pain radiating to lower limbs
2023-03-06	Acute illness consultation: lower limb weakness and right arm pain
2023-03-16	Acute illness consultation: paresthesia in the upper limbs
2023-04-19	Scheduled consultation with family physician (FP): seven-week history of insidious-onset and progressive tetraparesis, referral to neurology department
2023-05-04	Follow-up consultation with FP: impaired ability to use crutches, referral denied, requested Cervical CT scan and EMG
2023-05-24	Follow-up consultation with FP: findings in cervical CT scan and EMG lead to referral to the emergency department
2023-05-24 to 2023-06-06	Hospitalization in neurology department: underwent cervical and lumbar MRI, repeated EMG, established diagnosis
2023-06-21 to 2023-06-26	Elective hospitalization in neurosurgery department to perform artrodesis
2023-06-28	Scheduled consultation with FP: started physiotherapy treatments
2023-07-31, 2024-04-03	Subsequent neurosurgery consultations
2024-06-17	Last neurosurgery consultation: discharged

## Discussion

The family physician plays a central role in comprehensive healthcare, acting in the prevention, diagnosis, and treatment of multiple and varied diseases, and providing lifelong follow-up to the patient [14]. The role of the family doctor contributes to improving the population's quality of life and the efficiency of healthcare systems [14]. Furthermore, the general practitioner possesses broad and extensive knowledge of all organs and systems, allowing personalized care and a holistic approach encompassing physical, psychological, social, and spiritual aspects.

It is highly relevant to adopt a patient-centered approach, which meets patient expectations, provides comfort, treats symptoms and prevents complications [15]. Expectations must also be deconstructed if they are unrealistic, while always understanding and respecting the patient's sociocultural context [14]. This approach is fundamental to healthcare delivery, as it allows for the patient's active participation in healthcare decisions. It yields positive outcomes, including greater patient satisfaction, treatment adherence, and symptom reduction [15]. A systematic review and meta-analysis showed that individuals with acute and subacute LBP experience a notable and significant reduction in pain intensity and disability within an initial six-week period. However, those with persistent LBP had sustained high levels of pain and disability with minimal improvement over time. Consequently, clinical interventions should focus on identifying and escalating care in patients with subacute LBP who are exhibiting a slow recovery trajectory. This proactive approach aims to reduce the risk of transitioning to a chronic and persistent LBP with subsequent disability and low quality of life [16]. Patients presenting with low back pain require prompt screening for "red flag" clinical features that herald serious underlying spinal or neurological disease. Urgent assessment is necessary when symptoms include progressive neurological deficits, saddle anesthesia, new-onset bladder or bowel dysfunction, unexplained weight loss, or a history of recent trauma or malignancy. The early identification of these features is crucial for guiding immediate referral and subsequent diagnostic investigations [4].

The typical clinical course of acute and subacute low back pain is characterized by significant improvement in pain and disability within the first six weeks. However, in the present case, the persistence of symptoms and their divergence from the expected recovery pattern revealed a distinct and more serious underlying condition: compressive cervical myelopathy. This clinical mismatch highlights the importance of early recognition of red flags and atypical trajectories, which should prompt timely reassessment and exclusion of radiculomedullary pathology, particularly in patients with comorbidities that increase neurological vulnerability [17]. Cervical myelopathy is a progressive condition and remains the most common cause of spinal cord dysfunction in adults. Its presentation can be subtle in early stages, often masquerading as nonspecific musculoskeletal pain or peripheral neuropathy, leading to delays in diagnosis. Typical manifestations such as gait disturbance, hand clumsiness, upper extremity numbness, and hyperreflexia may initially be attributed to other comorbid conditions, especially in older patients or those with diabetes, as in the present case. Early identification is crucial, as neurological deterioration can become irreversible once significant cord compression or myelomalacia is established. Surgical decompression is the recommended approach and is associated with improved outcomes when performed early [17]. Thus, rather than focusing solely on low back pain, clinical evaluation should be directed toward identifying higher-level neurological etiologies when the clinical evolution deviates from the expected pattern.

Upper-limb symptoms may result from diverse etiologies, including peripheral neuropathy related to acute glycemic derangement and central causes such as degenerative cervical myelopathy (DCM). Recent studies have reinforced that MRI findings, particularly intramedullary T2-weighted hyperintensity, carry prognostic value in DCM: focal T2 hyperintensity, especially when accompanied by T1 hypointensity, and longer symptom duration or more severe pre-operative neurological deficit are associated with worse outcomes and higher risk of progression [18]. Quantitative MRI techniques (e.g., automated T2-signal analysis and diffusion tensor imaging metrics) have been shown to improve objectivity and may predict deterioration better than visual T2 changes alone. The correlation between clinical findings with conventional and quantitative MRI markers (T2 hyperintensity pattern, presence of T1 hypointensity, multilevel compression, and DTI-derived measures) is essential to guide diagnosis and management [18].

In this case, the final diagnoses were established as tetraparesis secondary to compressive cervical myelopathy and a sensorimotor polyneuropathy of predominantly axonal etiology. This polyneuropathy is likely of diabetic etiology and possible alcoholic contribution. This case could be compatible with diabetic amyotrophy, given the sudden onset of neuropathic pain and weakness and the typical patient profile, predominantly men generally over 50 years of age. Upper limb involvement is possible, and it can also result from an acute reduction in glycemic control [12]. Nevertheless, this condition typically starts with asymmetric or unilateral pain, whereas in this case the pain was reported as bilateral. The most recent EMG documented a moderate-to-severe chronic sensorimotor polyneuropathy with axonal predominance, which can occur not only in this condition but also in other types of diabetic neuropathy [12]. It was important to exclude compressive or infiltrative etiologies, which were achieved by performing neuroimaging. CT scan and MRI confirmed cervical discovertebral degenerative changes with potential radicular compromise. Surgical correction was performed, which resulted in an improvement of the upper limb symptoms. 

Syndromes often present with similar signs and symptoms, and a conclusive diagnosis is sometimes not possible. With each passing day, new markers and diagnostic methods are added to the medical arsenal, allowing for greater diagnostic accuracy and, consequently, more targeted and appropriate treatment for each patient. However, the pursuit of a diagnosis should never override the patient's comfort and needs. In this specific case, there is an overlap of multiple symptoms leading to a list of several possible diagnoses. The patient also has multiple comorbidities that, in themselves, contribute to a multiplicity of diagnostic biases. In fact, in the search for a single diagnosis, multiple may be identified. At times, despite a thorough investigation, a conclusive diagnosis may not be reached.

This case underscores the importance of a cautious approach to low back pain in a patient with a history of diabetes and alcohol use disorder, as these comorbidities can contribute to an increased susceptibility to neurological symptoms and disease [19]. It is important to check for 'red flags' and provide a timely diagnosis. Despite MRI being the recommended imaging approach, in the country where this case took place, general practitioners/family doctors can't prescribe MRI through the national healthcare system. A CT scan is free of charge, while an MRI is paid entirely by the patient, so the decision is often shared between the doctor and the patient, considering the patient's financial capacity. This aspect should be taken into consideration in potential future healthcare policies.

Given the persistence of the patient's complaints, an earlier assessment by the patient’s family physician should have been made. In this case, due to organizational constraints, it was done in the fourth consultation, seven weeks after the symptom onset. Also, the initial referral request was declined by the hospital, resulting in a delay in performing the MRI. Instead, a CT scan was mandated first. This requirement for a preliminary CT may have been detrimental or redundant, as MRI is the diagnostic modality with superior acuity for this specific clinical presentation.

It is essential to maintain a critical mindset and revise the differential diagnoses throughout the course of symptomatology. When uncertainty arises, one should question the current assessment, consider the re-evaluation, and, potentially, repeat prior diagnostic interventions. The initial EMG suggested a possible demyelinating component within EFNS/PNS criteria. However, axonal features were limited at that stage. Given the diagnostic overlap between GBS/CIDP, early diabetic neuropathy, and mixed toxic-metabolic neuropathies, a repeat EMG was performed during hospitalization. This second study demonstrated a chronic sensorimotor polyneuropathy with clear axonal predominance, aligning more closely with diabetic neuropathy and possible toxic-alcoholic contribution. This finding, combined with the patient’s medical history (notably without prior infections), physical examination, clinical evolution, and overall context, helped establish the most likely final diagnosis.

It was found that the decision to treat one of the identified pathologies (compressive cervical myelopathy) provided significant symptomatic relief for this patient, allowing him to regain autonomy and attain a higher quality of life. The subsequent Neurosurgery follow-up, physical rehabilitation treatments, managing cardiovascular risk factors, and mental health by the family doctor were essential to maintain and even achieve better results. However, despite the efforts made, the patient remained with some limitations, which led to his disability retirement. These changes in the patient's life had an impact on his psychological, financial, and social well-being. A systematic review from 2023 states that depressive, distress, and anxiety symptoms could be associated with poorer health outcomes in people with neck pain with or without radiculopathy [20]; therefore, it is crucially important to integrate mental health care throughout the clinical course of the disease.

Nevertheless, the work is not yet complete, and the activity of a family physician never truly ends. Each patient is unique and presents a continuum of interrelated symptoms and pathologies. The importance of the family physician in the management of complex patients and their correct guidance is of paramount significance.

## Conclusions

This case report demonstrates that common symptoms like lumbosciatalgia require an individualized approach, as they may mask complex conditions such as cervical myelopathy and polyneuropathy. Early identification of warning signs is critical; nevertheless, organizational limitations within the healthcare system delayed the necessary assessment and diagnostic imaging for this patient. By synthesizing signs from multiple consultations and reviewing the clinical history, the family physician was able to identify the underlying differential diagnoses. Subsequent multidisciplinary collaboration between family medicine, neurology, neurosurgery, and physical rehabilitation facilitated the patient's recovery. This report urges healthcare managers to revise healthcare policies, and it reinforces the family doctor's fundamental role in comprehensive management and 'red flag' identification, guaranteeing high standards of care. A persistent focus on holistic patient assessment and multidisciplinary collaboration is essential for overcoming systemic limitations, ensuring timely, accurate diagnosis of complex conditions hidden by common symptoms​​​​​​.
